# 

**Published:** 1894-09

**Authors:** 


### Communicated.

### AMERICAN DENTAL SOCIETY OF EUROPE.

The 19th annual meeting was held at Geneva, Aug. 6th to 8th. The following officers were elected for the ensuing year. President, Dr. Chas. W. Jenkins, Zurich; Vice-President. Dr. Wm. Mitchell, London; Treasurer, Dr. Chas. J. Monk, Wiesbaden; Secretary, Dr. Wm. S. Davenport, Paris; Executive Committee, Dr. Jenkins, Drs. G. C. Daboll and A. C. Hugenschmidt, of Paris; Membership Committee, Dr. Mitchell, Dr. L. C. Bryan of Basel, Dr. Waldo Royce, of Tunbridge Wells.

The next meeting will be held at Boulogne the first Monday in August 1895.

WM. S. DAVENPORT.

### To Readers and Correspondents.

Communications intended for publication, and exchange journals and papers, should be addressed to the Editor of The American Journal of Dental Science, No. 845 N. Eutaw St. Baltimore, Md. All letters relating to the business of the Journal, or containing cash remittances, to be directed to Messrs. Snowden & Cowman, No. 9 W. Fayette Street, Baltimore, Md. Articles for publication should be received by the Editor at least two weeks previous to the first of the month on which the number in which it is designed for them to appear, is issued.

☞ The American Journal of Dental Science will contain fifty pages of reading matter in each number, making a volume of about Six Hundred pages,

EXCLUSIVE OF ADVERTISEMENTS

